# Business Models and Profitability of Energy Storage

**DOI:** 10.1016/j.isci.2020.101554

**Published:** 2020-09-11

**Authors:** Felix Baumgarte, Gunther Glenk, Alexander Rieger

**Affiliations:** 1 FIM Research Center, University of Bayreuth, Project Group Business & Information Systems Engineering, Fraunhofer FIT, Bayreuth 95444, Germany; 2 Business School, University of Mannheim, MIT CEEPR, Massachusetts Institute of Technology, Mannheim 68131, Germany; 3 Interdisciplinary Centre for Security, Reliability and Trust, University of Luxembourg, Luxembourg 1855, Luxembourg

**Keywords:** Energy Management, Energy Storage, Energy Systems

## Abstract

Rapid growth of intermittent renewable power generation makes the identification of investment opportunities in energy storage and the establishment of their profitability indispensable. Here we first present a conceptual framework to characterize business models of energy storage and systematically differentiate investment opportunities. We then use the framework to examine which storage technologies can perform the identified business models and review the recent literature regarding the profitability of individual combinations of business models and technologies. Our analysis shows that a set of commercially available technologies can serve all identified business models. We also find that certain combinations appear to have approached a tipping point toward profitability. Yet, this conclusion only holds for combinations examined most recently or stacking several business models. Many technologically feasible combinations have been neglected, indicating a need for further research to provide a detailed and conclusive understanding about the profitability of energy storage.

## Graphical Abstract

**Figure:**
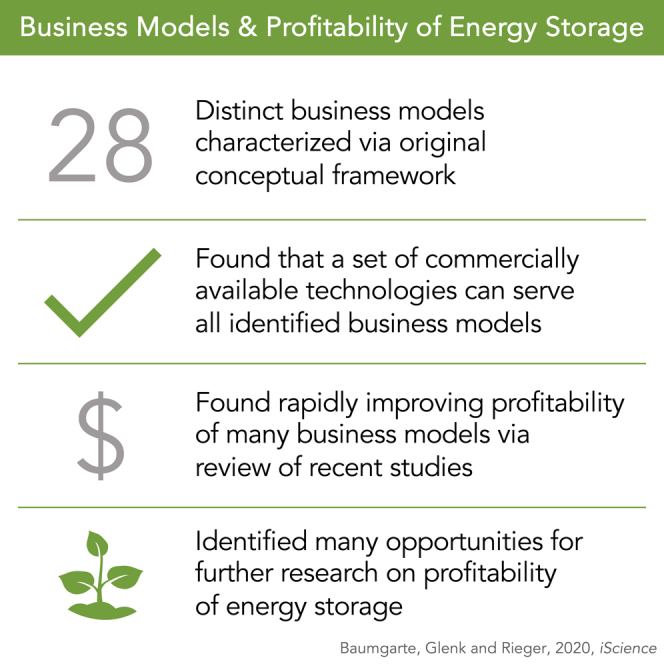

## Introduction

As the reliance on renewable energy sources rises, intermittency and limited dispatchability of wind and solar power generation evolve as crucial challenges in the transition toward sustainable energy systems (Olauson et al., 2016; Davis et al., 2018; Ferrara et al., 2019). Since electricity storage is widely recognized as a potential buffer to these challenges (Fares and Webber, 2017; Kittner et al., 2017; Davies et al., 2019), the number of advancements in energy storage technology and the amount of deployed capacity have rapidly grown in recent years (Schmidt et al., 2017; Comello et al., 2018; Sutherland, 2019; Blanc et al., 2020). The profitability of investment opportunities for storage overall, however, has remained ambiguous, partially due to an incomplete identification of such opportunities in modern power systems (Argyrou et al., 2018; Albertus et al., 2020) and contradicting conclusions about the profitability of individual opportunities (Braff et al., 2016; Kaschub et al., 2016; Fares and Webber, 2017; Metz and Saraiva, 2018; Comello and Reichelstein, 2019).

Numerous recent studies in the energy literature have explored the applicability and economic viability of storage technologies. Many have studied the profitability of specific investment opportunities, such as the use of lithium-ion batteries for residential consumers to increase the utilization of electricity generated by their rooftop solar panels (Hoppmann et al., 2014; Stephan et al., 2016; van der Stelt et al., 2018). Others have reviewed the range of potential applications of storage technologies, that is, the services that storage facilities can perform in power systems (Koohi-Kamali et al., 2013; Kousksou et al., 2014; Palizban and Kauhaniemi, 2016). Building upon both strands of work, we propose to characterize business models of energy storage as the combination of an application of storage with the revenue stream earned from the operation and the market role of the investor. Such business models can then be used to systematically differentiate investment opportunities, to assess which storage technologies are capable of serving a business model, and to review the profitability of individual combinations of business models and technologies.

This paper presents a conceptual framework to describe business models of energy storage. Using the framework, we identify 28 distinct business models applicable to modern power systems. We match the identified business models with storage technologies via overlaps in operational requirements of a business model and operational capabilities of a technology. The matching shows that all business models can be served by a set of commercially available technologies. Reviewing the results of previous studies on the profitability of individual matches, we find that they are largely found to be unprofitable. Yet, matches assessed since 2017 or comprising multiple business models served by one storage facility appear to have approached a tipping point toward profitability. Overall, our review reveals many technologically feasible matches that have been neglected so far. Their examination over the coming years will be essential to reach a detailed and conclusive evaluation of the profitability of energy storage. To conclude, we summarize the main research directions recommended in the reviewed literature to foster widespread profitability of storage.

## Results

### Business Models

We propose to characterize a “business model” for storage by three parameters: the application of a storage facility, the market role of a potential investor, and the revenue stream obtained from its operation (Massa et al., 2017). An application represents the activity that an energy storage facility would perform to address a particular need for storing electricity over time in modern power systems. A market role of potential investors refers to their assumed position in the electricity value chain. The revenue stream describes the type of income a storage facility can generate from its operation.

Table 1 provides a list and description of eight distinct applications derived from previous reviews on potential applications for energy storage (Castillo and Gayme, 2014; Kousksou et al., 2014; Palizban and Kauhaniemi, 2016). In the first three applications (i.e., provide frequency containment, short-/long-term frequency restoration, and voltage control), a storage facility would provide either power supply or power demand for certain periods of time to support the stable operation of the power grid. The following two applications in Table 1 (i.e., provide black start energy and backup energy) would support the availability of electricity at all times through the provision of power supply during blackouts either to reboot grid operations or to bridge the power outage for an electricity consumer. These five applications are frequently referred to as applications for ancillary services (Fuchs et al., 2012; Richter, 2013).

**Table 1:** Applications for Energy Storage

Application	Description
1) Provide frequency containment	Storage can stabilize the frequency and voltage of power supply providing either frequency containment, short- and long-term frequency restoration (The European Commission, 2017), or reactive energy for voltage control
2) Provide short-/long-term frequency restoration	
3) Provide voltage control	
4) Provide black start energy	Storage can support black starts of the electricity grid after a power outage and provide backup energy to bridge a power outage
5) Provide backup energy	
6) Meet selling/buying forecast	Storage can help meeting committed forecasts, adding power supply/demand when needed, for instance, during periods of unforeseen changes to the demand/generation profile
7) Shave supply/demand peaks	Storage can smooth out supply/demand curves and shave peaks
8) Sell at high/buy at low prices	Storage can improve power trades by buying at low and selling at high prices, including the utilization of surplus power from an onsite renewable energy source

The remaining three applications in Table 1 can be referred to as applications for load shifting as they focus on shifting electricity across time. In application (6) of Table 1, an energy storage facility would help meeting a committed selling/buying forecast, for instance, by compensating unforeseen changes in a demand or generation profile. In application (7), energy storage would shave supply/demand peaks and, for instance, avoid the expansion of transmission lines by reducing the peak of supply/demand in a particular geographic area. In application (8), the owner of a storage facility would seize the opportunity to exploit differences in power prices by selling electricity when prices are high and buying energy when prices are low.

As for the market role, we differentiate between the four main roles in the electricity value chain: trading, production, transmission and distribution (T&D), and consumption (Zucker et al., 2013). In trading, the investor would buy electricity from producers or the market and sell it to consumers or the market. In production, the investor would generate and sell electricity. In T&D, the investor is responsible for the transportation of electricity and the stable operation of the power grid. We aggregate the roles of a transmission and a distribution grid operator, because they appear compatible for the purpose of our study. Finally, an investor in consumption would purchase and consume electricity. We note that our concept of market roles is not equivalent to common descriptions of individual persons or organizations participating in the electricity market, even though they may coincide. An investor, that is, a person or an organization, can obtain multiple different roles or assume one role several times, for instance, by bundling consumers or producers, similar to how utilities and aggregators operate today. The decision to invest in a storage facility remains specific to each market role. Regulation is also often tied to market roles, potentially prohibiting the pursuit of distinct business models, as we review in the Discussion. T&D operators, for instance, are in many jurisdictions not allowed to provide frequency containment or restoration services.

For revenue streams, we delineate three different types, each comprising a range of distinct revenue streams. With “price arbitrage,” we refer to the utilization of differentials in electricity prices across markets at one time or across time within one market. The former can result from transaction costs, such as taxes and fees, which add to the market price when electricity is purchased rather than sold. For instance, residential consumers are typically paid less for electricity they produce with their solar panels and feed into the grid than they pay for sourcing electricity from the grid. The latter price differential results from fluctuations in electricity prices over time. “Cost avoidance” describes savings in operating costs, such as the ramping of power generation capacity, or penalties for, say, deviations in electricity production. Cost avoidance also includes savings in operating costs for electricity consumers, such as the ramping of a production facility for an industrial consumer or simply the inconvenience of changing behavior for a residential consumer. Finally, “investment deferral” refers to savings resulting from not investing in alternative generation or grid capacity.

**Figure 1:**
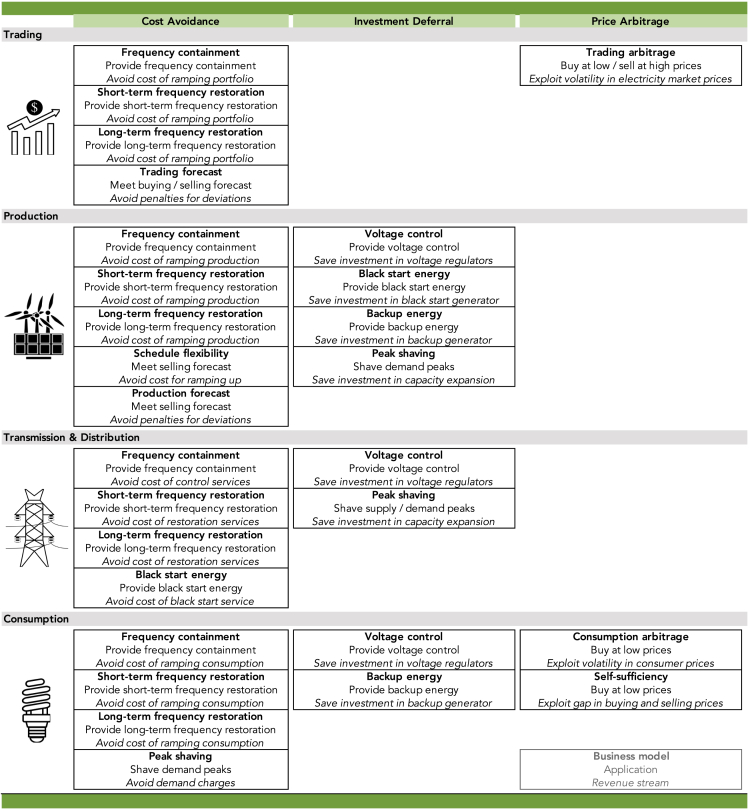
Business Models for Energy Storage Rows display market roles, columns reflect types of revenue streams, and boxes specify the business model around an application.

Figure 1 depicts 28 distinct business models for energy storage technologies that we identify based on the combination of the three parameters described above. Each business model, represented by a box in Figure 1, applies storage to solve a particular problem and to generate a distinct revenue stream for a specific market role. We determine the business models to be both mutually exclusive and collectively exhaustive. The former means that the business models are distinct from each other. The latter describes that we seek to record all observable and conceivable business models for a modern power system, recognizing that the identified set may change in the future.

Each of the three parameters is useful to systematically differentiate investment opportunities for energy storage in terms of applicable business models. The application as the central element defines what a storage facility would do in a business model. The application parameter is especially relevant if separate business models exhibit the same market role and the same revenue stream, such as for the business models we named (in bold letters in Figure 1) *Frequency containment, Short-term frequency restoration,* and *Long-term frequency restoration*.

Market roles are crucial for business models where the same application applies to several roles and generates the same revenue stream. All three frequency-related applications help the four market roles avoid costs. Market participants in trading, production, or consumption avoid the respective costs of ramping their portfolio, production, or consumption. Operators of a T&D grid would avoid costs of the control/restoration services offered by other market participants, provided they are allowed to do so by regulation. If an investor, that is a person or an organization, wants to provide one or more frequency-related applications simply for the price paid for this service, the investor would effectively pursue the business model *Trading arbitrage*. As the names suggest, *Trading/Consumption arbitrage* apply to trading and consumption, where energy storage enables the respective investor to sell at high prices and/or buy at low prices to take advantage of temporal fluctuations in electricity market prices. A version of price arbitrage may intuitively be assumed to also apply to producers, but they would then effectively act as traders and pursue the business model *Trading arbitrage*.

The business model *Voltage control* can apply to production, T&D, or consumption (Akhil et al., 2013), where the investment in energy storage would save the investment in a voltage regulator. Need for *Backup energy* typically arises at either the level of production or the level of consumption, where an energy storage facility would replace a conventional backup generator commonly based on diesel fuel. The meeting of forecasts applies to traders, who are obliged to purchase or sell a forecasted and contracted amount of electricity (i.e., *Trading forecast)*, as well as to producers, who have to deliver a contracted amount of power (i.e., *Production forecast*). Investment in energy storage can enable them to meet the contracted amount of electricity more accurately and avoid penalties charged for deviations.

Revenue streams are decisive to distinguish business models when one application applies to the same market role multiple times. *Schedule flexibility* and *Production forecast* both help an investor in production to meet a selling forecast. Yet, the former avoids the cost of ramping the production capacity, whereas the latter avoids penalties charged for deviations from the forecast. Similarly, *Consumption arbitrage* and *Self-sufficiency* allow an investor in consumption to buy more electricity during periods of low prices. The former takes advantage of fluctuations in power prices over time, whereas the latter exploits that selling prices for electricity generated with own renewable sources are at times below the buying prices for electricity sourced from the grid.

Market roles and revenue streams may also jointly differentiate business models. *Black start energy* can be pursued by an investor in production, who seeks to defer the investment in a black start generator with an investment in energy storage. Alternatively, the business model can be pursued by an investor in T&D, who seeks to avoid or lower costs of sourcing black start services through a competitive tender if market regulation permits (Denholm et al., 2010). The business model *Peak shaving* can be pursued by an investor in production, T&D, or consumption. For the former two energy storage can defer the investment in production or transmission capacity, whereas for the latter storage lowers charges by utilities for periodical demand peaks.

The literature on energy storage frequently includes “renewable integration” or “generation firming” as applications for storage (Eyer and Corey, 2010; Zafirakis et al., 2013; Pellow et al., 2020). Yet, for storage combined with a dispatchable power generator, such as a gas turbine, the terms describe *Schedule flexibility* to avoid the cost of ramping the generator up and down. For storage combined with renewables, the terms may describe the meeting of *Production forecasts* to avoid penalties for underproduction. Alternatively, the terms may describe *Trading arbitrage* if storage is installed to take advantage of excess production from wind and solar power sources, which can without storage be shut down at negligible cost. Similarly, the term “long-term storage” is reflected in the business models *Trading arbitrage*, *Black start energy*, *Backup energy*, or *Self-sufficiency*, depending on the actual implementation of the storage facility.

Investors can pursue multiple business models with a single storage capacity if market regulation permits. Applicable examples for business models that are frequently combined include the combination of *Frequency containment* with *Frequency restoration*, the combination of *Consumption arbitrage* with *Self-sufficiency*, or the combination of *Frequency containment* with *Trading arbitrage* (Stephan et al., 2016; Berrada et al., 2017; Yu and Foggo, 2017).

### Profitability

We now use the preceding framework to systematically review recent studies on energy storage regarding their findings on the profitability of potential investments. Our goal is to give an overview of the profitability of business models for energy storage, showing which business model performed by a certain technology has been examined and identified as rather profitable or unprofitable. We refrain from attempting to compare specific investments, which depend on regionally distinct economic, operational, and regulatory parameters.

Before providing the profitability overview, we first examine whether a technology has the capability to serve a business model. Each business model entails specific operational requirements through its application, but each technology can only operate within distinct ranges. We match the business models identified above to a set of technologies via overlaps in operational parameters that we extracted from technical reports as well as previous reviews and technology-specific articles in peer-reviewed journals (Schoenung, 2001; EPRI, 2003, 2010; Barton and Infield, 2004; Eyer et al., 2004; McDowall, 2006; Sayer et al., 2007; Chen et al., 2009; Eyer and Corey, 2010; Beaudin et al., 2010; Connolly, 2010; Denholm et al., 2010; Akhil et al., 2013; Koohi-Kamali et al., 2013; Del Rosso and Eckroad, 2014; Palizban and Kauhaniemi, 2016; Eid et al., 2016). We examine the parameters power capacity, discharge duration, and response time. These reflect non-negotiable requirements for business models. Details on the matching and the used parameters are provided in the Supplemental Information.

We focus on a set of common and commercially available technologies for energy storage (see Table S1 for details). These technologies convert electrical energy to various forms of storable energy. For mechanical storage, we focus on flywheels, pumped hydro, and compressed air energy storage (CAES). Thermal storage refers to molten salt technology. Chemical storage technologies include supercapacitors, batteries, and hydrogen. Of the various battery technologies available, we focus on lithium-ion batteries, which have recently exhibited the most rapid cost declines and technological advances (Schmidt et al., 2017).

In comparison, flywheels have a medium power capacity and can respond spontaneously but commonly discharge in less than an hour. Pumped hydro and CAES currently offer the largest power capacity and a sustained discharge duration but require several minutes to respond as well as appropriate geographic formations. Thermal storage responds within minutes and exhibits a medium power capacity with discharge durations of several hours. Supercapacitors can respond instantly but frequently display the smallest power capacity and discharge duration. Batteries show a medium power capacity range and discharge duration and a short response time. Finally, hydrogen storage can have a relatively large power capacity with a long discharge duration but requires several minutes to respond from a cold start (see Tables S2 and S3 for details).

To depict the quality of a match, we employ a simple traffic light scheme. We consider a match as “green” if the capabilities of a technology overlap with the requirements of a business model in all three characteristics. Alternatively, a match is “yellow” if the parameters overlap in only two characteristics and “red” if they overlap in one or none. This simple scheme only provides a snapshot of the current development but is helpful to quickly grasp the quality of a match.

**Figure 2:**
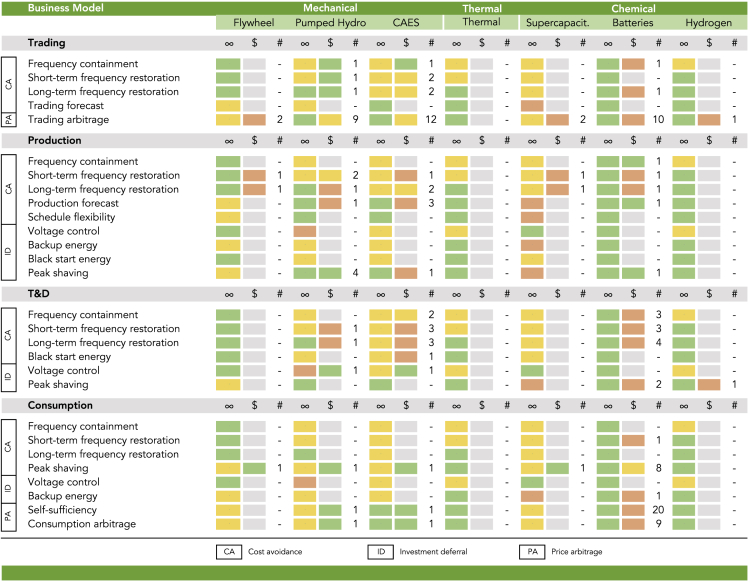
Technology Match and Profitability of Business Models for Energy Storage The first column (∞) indicates the matching of business models with storage technologies, the second column ($) the profitability, and the third column (#) the number of studies that examine the profitability of a match.

Figure 2 shows for each technology in the first column the result of this matching. We find that every business model can be served (i.e., green match) by at least one of the commercially available storage technologies and that most business models can even rely on multiple technologies. The matching confirms the widespread preference of batteries and hydrogen in the sense that these technologies can serve almost all business models. Yet, the matching also highlights many green matches for other technologies, such as flywheels and thermal storage. CAES is green for only a few matches, such as *Self-sufficiency* and *Consumption arbitrage*, noting that the market role also includes large industrial consumers. Pumped hydro is often either too slow to respond (e.g., for frequency containment and short-term restoration) or too large in its minimal power capacity (e.g., for consumption). Supercapacitors often fall below the required power capacity and discharge duration. The matching assumes that business models in Figure 2, which entail the same application, have the same range of operational requirements.

For economic opportunities, we aim at extracting a similar map. Our review is based on 143 profitability estimates for individual business model and technology combinations. The estimates result from a systematic literature review of articles in peer-reviewed journals from 2013 to 2019 with selected keywords. Since our objective is to identify general opportunities for storage rather than evaluating distinct investment cases, we aggregate estimates across valuation methodologies and geographical parameters. To ensure quality, applicability, and comparability, we narrowed down the set of 489 articles initially retrieved from the review to 47 focus papers with several criteria, including the ranking of the journal, the rigor of the analysis, as well as the comparability of the research setting (see the Supplemental Information for details).

We again use the traffic light scheme to illustrate profitability estimates of each match. We consider a match as green if the share of estimates that finds the match to be profitable is above 75%. Similarly, a match is yellow if the share of profitable estimates is between 50% and 75% and red if the share is below 50%. In addition, we label a match as “gray” if our review returned no estimate for the match. More optimistic color thresholds would not change the overall conclusion. A figure with numerical results is provided in Figure S1 in the Supplemental Information. For a sense of confidence in our findings, we also report the number of profitability estimates for the respective finding. Across all matches, the number of estimates also indicates the distribution of research effort.

Figure 2 shows the result of the profitability review in the second and third column of each technology. The main finding is that examined business models for energy storage given in the set of technologies are largely found to be unprofitable or ambiguous. Our finding is corroborated by both the distribution of profitability labels in Figure 2 (31 red, 8 yellow, and 18 green) and the average number of estimates per profitability label (2.7 for red, 4.9 for yellow, and 1.2 for green). This conclusion applies in particular to batteries (13 of 17 examined business models are red), which opposes the image of a promising complement to intermittent renewable power sources. The technology with the highest number of green profitability labels (i.e., 8) is pumped hydro. New installations of pumped hydro, however, are often limited by either the availability of caverns and mountains or public resistance to environmental changes.

Figure 2 also delineates that research on the profitability of energy storage is distributed unevenly across technologies, business models, and matches. The by far most examined technologies are batteries (68 profitability estimates), CAES (37), and pumped hydro (26). The most prominent business models are frequency containment (44 profitability estimates for *Frequency containment* and *Short-* and *Long-term frequency restoration* combined), *Trading arbitrage* (36), and *Self-sufficiency* (22). The most examined matches also result from this pool and comprise batteries for *Self-sufficiency* (20 profitability estimates), and pumped hydro and CAES for *Trading arbitrage* (9 and 12 estimates). This distribution unveils a considerable potential for future research (71 of 139 gray labels have a green label for the technology match), in particular, for flywheels used for ancillary services and thermal and hydrogen storage in general.

Although academic analysis finds that business models for energy storage are largely unprofitable, annual deployment of storage capacity is globally on the rise (IEA, 2020). One reason may be generous subsidy support and non-financial drivers like a first-mover advantage (Wood Mackenzie, 2019). Another reason may be the time lag between the publication of academic articles and the market development. Some storage technologies have exhibited a substantial cost decline in recent years (Kittner et al., 2017; Schmidt et al., 2017; Glenk and Reichelstein, 2019). The cost of battery cells, for instance, decreased from above US$1,100/kWh in 2010 to less than US$156/kWh in 2019 (BNEF, 2019). Repeating our review with papers from 2017 to 2019 only, we find the conclusion to improve markedly, as shown in Figure S2 in the Supplemental Information. Of the 19 examined business models 14 are now green. Batteries contribute 6 green business models, of which 5 have flipped from red to green in comparison with Figure 2. These green business models include *Trading arbitrage, Production forecast,* as well as *Frequency containment/restoration* on a trading and T&D level*.* The residual green matches comprise pumped hydro and CAES for *Trading Arbitrage, Self-sufficiency,* and *Consumption arbitrage*, as well as pumped hydro for *Short-term restoration* and *Peak shaving* for the production level. Most of the green labels, however, rely on only few studies.

**Figure 3:**
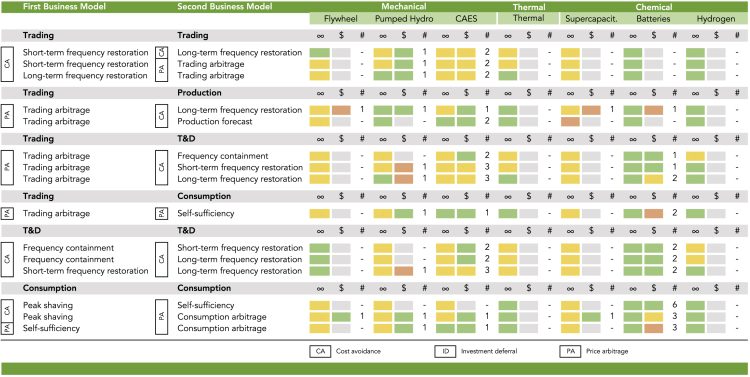
Technology Match and Profitability of Stacked Business Models The first column (∞) indicates the matching of business models with storage technologies, the second column ($) the profitability, and the third column (#) the number of studies that examine the profitability of a match.

A third reason may be the stacking of business models. Stacking describes the simultaneous serving of two or more business models with the same storage unit (Schmidt et al., 2017). This can allow a storage facility to both diversify its revenue streams and to increase its utilization by bridging idle time in one business model with operation in another. To assess the effect of stacking on profitability, we reviewed the focus papers again and collected the profitability estimates of matches with stacked business models. Figure 3 shows that the stacking of two business models can already improve profitability considerably. Of 39 stacks analyzed in the literature, 23 profitability labels are green, 8 are yellow, and 8 are red. The most frequent stacks are combinations of consumption business models, such as *Self-sufficiency* with *Consumption peak shaving*, combinations of frequency containment/restoration business models with *Trading arbitrage*, or aggregations of multiple frequency containment/restoration business models. The most examined technologies are again CAES (27 profitability estimates), batteries (25), and pumped hydro (10).

Recent deployments of storage capacity confirm the trend for improved investment conditions (U.S. Department of Energy, 2020). For instance, the Imperial Irrigation District in El Centro, California, installed 30 MW of battery storage for *Frequency containment*, *Schedule flexibility*, and *Black start energy* in 2017. The Deepwater Wind in Montauk, New York, built 15 MW of battery storage for *Production forecast* in 2018. The Hornsdale Power Reserve in Jamestown, South Australia, has been using grid-scale battery storage with a capacity of 100 MW for *Frequency containment* and *Peak shaving* since 2017. Nant de Drance in Martigny, Switzerland, is constructing 900 MW of pumped hydro storage for *Peak shaving* and *Production forecast* with a planned start of operations in 2021. A study by RWTH Aaachen has reported more than 120,000 residential PV battery systems in Germany by the end of 2018 with a cumulative capacity of 400 MW used for *Self-sufficiency* and *Consumption arbitrage*. Finally, the HyBalance project located in Hobro, Denmark, installed 2 MW of hydrogen storage for *Frequency restoration* and *Peak shaving* in 2017.

## Discussion

Although electricity storage technologies could provide useful flexibility to modern power systems with substantial shares of power generation from intermittent renewables, investment opportunities and their profitability have remained ambiguous. Here we first present a conceptual framework to characterize business models of energy storage and, thereby, systematically differentiate investment opportunities. Our framework identifies 28 distinct business models based on the integrated assessment of an application for storage with the market role of the potential investor and the achievable revenue stream from the storage operation. We then use our framework to match storage technologies with the identified business models and to review findings of previous studies on the profitability of individual matches. Our review shows that a set of commercially available technologies is sufficient to perform all identified business models. We also find that matches appear to have approached a tipping point toward profitability. Yet, this conclusion only holds for matches that either have been examined since 2017 or entail multiple business models. Overall, many feasible matches have been ignored, indicating research gaps that need to be filled for a detailed and conclusive understanding of the profitability of energy storage.

Widespread profitability of storage will also require continued work on incremental improvements in both technological and regulatory parameters of storage. Our focus papers highlight, in particular, the need for a reduction of the overall costs of storage technologies and the removal of revenue barriers in a business model. Since the overall costs of storage installations are largely upfront investment, continued declines in the acquisition cost of storage technology are of paramount importance (Madlener and Latz, 2013; Dufo-López and Bernal-Agustín, 2015; de Sisternes et al., 2016; Kaschub et al., 2016; Yu and Foggo, 2017; Hartmann et al., 2018). Reductions may primarily come from technological advancements, such as the use of cheaper materials, improved component architectures, or economies of scale in manufacturing (Comello and Reichelstein, 2019). An improved round-trip efficiency, cycle capacity, and lifetime can further reduce the overall costs (Madlener and Latz, 2013; Dufo-López and Bernal-Agustín, 2015; Lai and McCulloch, 2017; Yu and Foggo, 2017; Chazarra et al., 2018). These characteristics increase the degree of utilization and reduce the amount of costly capacity required for a storage project.

Revenue gains can result from the creation of innovative support schemes and the removal of regulatory barriers. Such support schemes could ensure effectiveness by using our conceptual framework and its parameters. With the market role as one crucial parameter, multiple vested interests could be addressed. One example of how this could be achieved is the public tender for the later Hornsdale Power Reserve. The tender combined interests of the T&D operator by including a certain capacity that was to be contracted to save investments in capacity expansion and interests of an investor by embracing a trading role to use the remaining capacity for exploitation of volatility of market prices (Australian Energy Market Operator, 2018). The revenue stream parameter allows one to differentiate the type of support mechanisms. Where a profitable application of energy storage requires saving of costs or deferral of investments, direct mechanisms, such as subsidies and rebates, will be effective. For applications dependent on price arbitrage, the existence and access to variable market prices are essential.

Prominent regulatory barriers include limited market access for energy storage (Castagneto Gissey, Dodds and Radcliffe, 2018), bans on stacking business models (Stephan et al., 2016), and regulatory markups on electricity prices (Reuter et al., 2012; Mulder et al., 2013; Bradbury et al., 2014; Khalilpour and Vassallo, 2016; Shafiee et al., 2016; Berrada et al., 2017; Lin and Wu, 2017). The recent FERC Order 841 in the Unites States, for instance, reflects one of the first regulatory changes that entitle storage solutions to participate in wholesale power markets, which they are able to serve from a technical point of view (FERC, 2018). The order opens wholesale markets to smaller actors, compelling system operators to modify access requirements where possible and to include energy storage, for instance, through a smaller minimum capacity size.

Another area for policy reform is the stacking of business models, which is still banned in many jurisdictions (Stephan et al., 2016). The California Public Utilities Commission (CPUC) took a first step and published a framework of eleven rules prescribing when energy storage is allowed to provide multiple services. The framework delineates which combinations are permitted and how business models should be prioritized (American Public Power Association, 2018). Bolder approaches could include the design of special electricity tariffs for investors in a consumer role that unlock the ability of energy storage to mitigate unexpected demand peaks (*Peak Shaving*) and balance conventional demand patterns (*Consumption Arbitrage*) (Fridgen et al., 2018).

Moreover, regulators could revisit markups on wholesale electricity prices, such as taxes and fees, that may impede storage investments through the curtailment of available revenue streams in a jurisdiction. For instance, before the modification of the Renewable Energy Act in 2017, storage facilities in Germany were considered as final consumers and, consequently, paid all regulatory price markups for the electricity used for charging (EEG, 2017; Glenk and Reichelstein, 2020).

### Limitations of the Study

The identified business models are a snapshot of present economic opportunities in the energy sector and could change over the years coming. Especially with regard to future changes within modern power systems, the identified business models may no longer be mutually exclusive and collectively exhaustive. Moreover, we reviewed only a representative sample of the available literature to extract the profitability of business models applicable to modern power systems. Thus, some matches may not have received a profitability validation that reflects their present profitability. We hope, nevertheless, that our approach may be a foundation for future economic analyses and fosters comparability for future findings about economic opportunities of energy storage.

## Methods

All methods can be found in the accompanying Transparent Methods supplemental file.

## Supplemental Information

Document S1. Transparent Methods, Figures S1 and S2 and Tables S1–S4

## Footnotes

Supplemental Information can be found online at https://doi.org/10.1016/j.isci.2020.101554.
