# Supplementary material for: Business Models and Profitability of Energy Storage
Source: iScience. 2020 Sep 11;23(10):101554. doi: 10.1016/j.isci.2020.101554 (PMC7528196; doi:10.1016/j.isci.2020.101554)
Supplement: Document S1. Transparent Methods, Figures S1 and S2 and Tables S1–S4 [file mmc1.pdf]

iScience, Volume *23*

Supplemental Information

Business Models and Profitability of Energy Storage

Felix Baumgarte, Gunther Glenk, and Alexander Rieger

Transparent Methods

Matching

To match the described business models with a set of commercially available technologies, we look for an overlap in three operational parameters. Power capacity indicates the peak amount of power (in megawatt) a storage device should be able to absorb or provide at any time. Discharge duration (in hours) denotes the time a storage device should provide electricity at peak power capacity and effectively reflects the amount of stored energy. Response time (in seconds) measures the speed with which a storage technology should react to a charging or discharging request.

Some studies also use the cycle capacity, round-trip efficiency, and self-discharge of storage technologies for matching (Aneke and Wang, 2016; Palizban and Kauhaniemi, 2016). Cycle capacity describes the number of full charging and discharging cycles a storage device can perform until it reaches a specified degradation level. Round-trip efficiency defines the amount of energy recoverable from a storage device relative to the amount initially absorbed. Self-discharge labels the amount of charged energy lost during inactivity. Contrary to these studies, we discourage the use of these parameters for the matching of functionality, as in serving a business model they rather affect a technology's cost efficiency.

For each combination, the capability of a technology T must match with the requirements of a business model BM. First, the storage technology's power capacity range must overlap with the required power capacity range of the business model. In particular, the storage technology must have a maximum power capacity C_T^{max} greater than or equal to the minimum required capacity C_{BM}^{min} of the respective business model. At the same time, the technology's minimal possible storage capacity C_T^{min} must be less than or equal to the maximum required capacity C_{BM}^{max} of the business model:

$$C_T^{max} \geq C_{BM}^{min} \quad \text{and} \quad C_T^{min} \leq C_{BM}^{max} \tag{1}$$

The storage technology must also have a maximum discharge duration D_T^{max} that is longer than or equal to the shortest required discharge duration D_{BM}^{min} of the business model:

$$D_T^{max} \geq D_{BM}^{min} \tag{2}$$

Further, the storage technology must have a minimum response time R_T^{min} that is smaller than or equal to the slowest required response time R_{BM}^{max} of the business model:

$$R_T^{min} \leq R_{BM}^{max} \quad (3)$$

Our analysis focuses on a set of commercially available technologies. Supplemental Table S1 provides a list and description of these technologies. Supplemental Tables S2-S3 outline the operational requirements of business models and operational capabilities of storage technologies used for the matching. The parameters were collected from various sources as indicated for each parameter.

Profitability Review

We systematically reviewed scientific articles in peer-reviewed journals and frequently cited grey literature such as reports by reputable agencies, consultancies, and industry analysts. We found the initial set of articles by searching the databases ScienceDirect, IEEE, EBSCOhost, and Google Scholar with a combination of various keywords including specific energy storage technologies (e.g. "flywheel"), business models (e.g. "frequency containment"), general keywords (e.g. "energy storage"), and keywords related to profitability assessment (e.g. "valuation"). We then traced citations in the set of articles backward or citations to the set of articles forward to maximize the coverage of relevant articles.

We retrieved 489 articles and reports, which we narrowed down through the application of several criteria to control for quality and fit. We first excluded all reports as well as articles published before 2012 or in journals with a rank below 1.0 in the Scimago Journal and Country Rank to ensure quality and timeliness of our analysis. We then filtered the remaining papers for individual and stacked profitability analyses based on their title and abstract. This resulted in a set of 47 focus papers, most of which were published in the last three years.

For every focus paper we first extracted general information (i.e. technology and market setting), as well as the applications and business models that were analyzed. We then examined all focus papers regarding their findings on single and stacked profitability and the assumptions made. Finally, we collected what the authors have identified as the main opportunities and barriers for the emergence of profitable business models for storage, including their suggestions for policy changes. A table of our focus papers is provided in Supplemental Table S4. Supplemental Figure S1 shows the result of the technology matching and the profitability review in numbers, on which the colored labels of Figure 2 of the main body are based. Supplemental Figure S2 shows the result of the profitability review based on studies from 2017-2019.

Supplemental Tables

Supplemental Table S1, Related to Figure 2 and Figure 3.

List and description of energy storage technologies.

Technology	Storage medium	Charging and discharging process
**Flywheels**	Rotating mass	Charging: an electric motor accelerates a rotatable mass that is held in place by magnets to minimize friction. Discharging: the rotating mass drives a generator.
**Pumped hydro**	Water reservoir at higher altitude	Charging: water is pumped uphill into a reservoir. Discharging: water flows back down and drives hydroelectric turbines.
**CAES**	Compressed air in tank or cavern	Charging: ambient air is pumped into storage tanks or cavernous rock formations. Discharging: pressurized air is released through a turbine.
**Thermal**	Heat stored as molten salt	Charging: heat pump heats tank with a solid salt that, once molten, is stored in a hot tank. Discharging: the molten, hot salt drives a steam turbine and flows back to the cold tank.
**Supercapacitors**	Separated charges	Charging: electric current creates a double layer of oppositely charged molecules of electrolyte on either side of an insulator. Discharging: electrons flow from one side of the insulator to the other and balance the charge gradient.
**Batteries**	Separated ions	Charging: electric current ionizes neutral molecules, which move to oppositely charged electrodes separated by an insulator. Discharging: electrons flow from the negative to the positive electrode, de-ionize molecules and balance the charge gradient.
**Hydrogen**	Hydrogen in tank, cavern or pipeline	Charging: electric current splits water molecules into oxygen and hydrogen. Discharging: hydrogen is burnt in gas turbines or recombined with oxygen in a (reversible) fuel cell.

Supplemental Table S2, Related to Figure 2 and Figure 3.

Operational requirements of business models.

**Business Model**	**Power capacity [MW]**			**Discharge duration [h]**			**Response time [sec]**		
	min	max	source	min	max	source	min	max	source
**Trading**									
Frequency containment	1	100	[1]	0.25	1	[3]	0.001	15	[4]
Short-term frequency restoration	1	100	[1]	0.25	1	[3]	10	30	[3]
Long-term frequency restoration	1	100	[1]	1	2	[5]	30	60	[3]
Trading forecast	1	10	-	2	2	[6]	60	600	[9]
Trading arbitrage	40	400	[2]	1	10	[4]	60	3600	[10]
**Production**									
Frequency containment	1	100	[1]	0.25	1	[3]	0.001	15	[4]
Short-term frequency restoration	1	100	[1]	0.25	1	[3]	10	30	[3]
Long-term frequency restoration	1	100	[1]	1	2	[5]	30	60	[3]
Production forecast	1	100	[3]	2	4	[5]	60	3600	[10]
Schedule flexibility	1	500	[4]	2	8	[7]	60	1800	[4]
Voltage control	0.1	10	[4]	0.25	1	[5]	0.001	0.1	[4]
Backup energy	1	10	[4]	4	10	[1]	10	60	[4]
Black start energy	5	50	[3]	1	5	[8]	1	60	[10]
Peak shaving	1	500	[3]	4	8	[5]	60	1800	[4]
**T&D**									
Frequency containment	1	100	[3]	0.25	1	[3]	0.001	15	[4]
Short-term frequency restoration	1	100	[3]	0.25	1	[3]	10	30	[3]
Long-term frequency restoration	1	100	[1]	1	2	[5]	30	60	[3]
Black start energy	5	50	[3]	1	5	[1]	1	60	[10]
Voltage control	0.1	10	[4]	0.25	1	[5]	0.001	0.1	[4]
Peak shaving	1	100	[3]	2	6	[1]	60	3600	[10]
**Consumption**									
Frequency containment	1	100	[1]	0.25	1	[3]	0.001	15	[4]
Short-term frequency restoration	1	100	[1]	0.25	1	[3]	10	30	[3]
Long-term frequency restoration	1	100	[1]	1	2	[5]	30	60	[3]
Peak shaving	0.05	10	[5]	5	11	[5]	1	60	[11]
Voltage control	0.1	10	[4]	0.003	0.25	[5]	0.01	0.2	[4]
Backup energy	1	10	[4]	4	10	[1]	10	60	[4]
Self-sufficiency	0.001	1	[3]	2	6	[1]	60	3600	[10]
Consumption arbitrage	0.05	10	[5]	5	11	[5]	60	3600	[10]

Sources:

[1] (EPRI, 2010)
[2] (Koohi-Kamali *et al.*, 2013)
[3] (Akhil *et al.*, 2013)
[4] (Palizban and Kauhaniemi, 2016)
[5] (Eyer and Corey, 2010)
[6] (Barton and Infield, 2004)
[7] (Sayer, Eyer and Brown, 2007)
[8] (Eyer, Iannucci and Corey, 2004)
[9] (Beaudin *et al.*, 2010)
[10] (Denholm *et al.*, 2010)
[11] (Schoenung, 2001)

Supplemental Table S3, Related to Figure 2 and Figure 3.

Operational capabilities of storage technologies.

**Technology**	**Power capacity [MW]**			**Discharge duration [h]**			**Response time [sec]**		
	min	max	source	min	max	source	min	max	source
**Flywheels**	0.001	20	[1]	0.1	1	[6]	0.01	1	[2]
**Pumped hydro**	100	5000	[2]	6	24	[1]	60	180	[2]
**CAES**	1	400	[3]	1	24	[2]	180	600	[7]
**Thermal**	0.1	300	[3]	1	24	[3]	60	600	[1]
**Supercapacitors**	0.001	0.3	[4]	0.0027	1	[2]	0.001	0.1	[1]
**Batteries**	0.1	50	[2]	0.1	5	[3]	0.003	0.1	[7]
**Hydrogen**	0.1	50	[5]	1	24	[2]	30	300	[8]

Sources:

[1] (Palizban and Kauhaniemi, 2016)
[2] (Gallo *et al.*, 2016)
[3] (Ferreira *et al.*, 2013)
[4] (Chen *et al.*, 2009)
[5] (Aneke and Wang, 2016)
[6] (Cho, Jeong and Kim, 2015)
[7] (Fuchs *et al.*, 2012)
[8] (Bertuccioli *et al.*, 2014)

Supplemental Table S4, Related to Figure 2 and Figure 3. List and analysis of the focus papers.

Articles	Technology	Application	Business Model	Profitability	Stacking
(Arabkoohsar *et al.*, 2015)	CAES	Meet selling forecast (production); Sell at high prices (trading); Buy at low prices (trading);	Production forecast; Trading arbitrage	yes	yes
(Battke and Schmidt, 2015)	Batteries	Buy at low prices (consumption); Sell at high prices (trading); Buy at low prices (trading); Provide frequency containment (T&D); Provide short-term frequency restoration (T&D); Provide long-term frequency restoration (T&D); Provide backup energy (consumption)	Consumption arbitrage; Trading arbitrage; Frequency containment; Short-term frequency restoration; Long-term frequency restoration; Backup energy; Self-sufficiency	no	No
(Berrada and Loudiyi, 2016)	Pumped Hydro; Batteries; Flywheel; Supercaps; CAES	Sell at high prices (trading); Buy at low prices (trading); Provide frequency containment (production); Provide short-term frequency restoration (production); Provide long-term frequency restoration (production)	Trading arbitrage; Frequency containment; Short-term frequency restoration; Long-term frequency restoration	no	yes
(Berrada, Loudiyi and Zorkani, 2016)	Pumped Hydro; CAES; Gravity Storage	Provide frequency containment (trading); Provide short-term frequency restoration (trading); Provide long-term frequency restoration (trading); Provide voltage control (T&D); Sell at high prices (trading); Buy at low prices (trading)	Frequency containment; Short-term frequency restoration; Long-term frequency restoration; Voltage control; Trading arbitrage	yes	yes
(Berrada, Loudiyi and Zorkani, 2017)	Gravity Storage; CAES; Pumped Hydro; Batteries	Buy at low prices (consumption); Sell at high prices (trading); Buy at low prices (trading);	Consumption arbitrage; Trading arbitrage; Self-sufficiency	yes	yes
(Bortolini, Gamberi and Graziani, 2014)	Batteries	Buy at low prices (consumption)	Self-sufficiency	yes	no
(Bradbury, Pratson and Patiño-Echeverri, 2014)	Batteries; Supercaps; Pumped Hydro; CAES; Flywheel	Sell at high prices (trading); Buy at low prices (trading)	Trading arbitrage	yes	no
(Braff, Mueller and Trancik, 2016)	CAES; Pumped Hydro; Batteries	Sell at high prices (trading); Buy at low prices (trading)	Trading arbitrage	yes	no
(Broneske and Wozabal, 2016)	Batteries	Provide short-term frequency restoration (consumption)	Short-term frequency restoration	no	no
(Chazarra *et al.*, 2018)	Pumped Hydro	Provide short-term frequency restoration (production); Shave demand peaks (production)	Short-term frequency restoration; Peak shaving	yes	yes
(Comello and Reichelstein, 2019)	Batteries	Buy at low prices (consumption)	Self-sufficiency	yes	no
(Das, Krishnan and McCalley, 2015)	CAES	Provide frequency containment (T&D); Provide short-term frequency restoration (T&D); Provide long-term frequency restoration (T&D); Provide black start energy (T&D); Meet selling forecast (production); Sell at high prices (trading); Buy at low prices (trading)	Frequency containment; Short-term frequency restoration; Long-term frequency restoration; Production forecast; Black start energy; Trading arbitrage	no	yes
(de Bosio and Verda, 2015)	CAES	Buy at low prices (trading); Sell at high prices (trading); Provide frequency containment (T&D); Provide short-term frequency restoration (T&D); Provide long-term frequency restoration (T&D)	Frequency containment; Short-term frequency restoration; Long-term frequency restoration; Trading arbitrage	yes	yes
(de Sisternes, Jenkins and Botterud, 2016)	Pumped Hydro; Batteries	Shave demand peaks (production);	Generation capacity reserve;	yes	no

(Dufo-López and Bernal-Agustín, 2015)	Batteries	Buy at low prices (consumption)	Consumption arbitrage	no	no
(Fares and Webber, 2017)	Batteries	Buy at low prices (consumption)	Self-sufficiency	no	no
(Fleer *et al.*, 2018)	Batteries	Provide frequency containment (trading)	Frequency containment	no	no
(Gough *et al.*, 2017)	Batteries	Provide long-term frequency restoration (trading); Buy at low prices (consumption); Shave demand peaks (consumption)	Long-term frequency restoration; Consumption arbitrage; Self-sufficiency; Peak shaving	yes	yes
(Härtel *et al.*, 2016)	Batteries; Hydrogen	Shave supply / demand peaks (T&D)	Peak shaving	no	no
(Hartmann, Divényi and Vokony, 2018)	Batteries	Shave demand peaks (consumption); Buy at low prices (consumption)	Peak shaving; Consumption arbitrage	no	no
(Hoppmann *et al.*, 2014)	Batteries	Buy at low prices (consumption)	Self-sufficiency	yes	no
(Johnston *et al.*, 2015)	Batteries	Provide frequency containment (production)	Frequency containment	yes	no
(Kaschub, Jochem and Fichtner, 2016)	Batteries	Buy at low prices (consumption); Shave demand peaks (consumption)	Self-sufficiency; Peak shaving	yes	yes
(Khalilpour and Vassallo, 2016)	Batteries	Buy at low prices (consumption)	Self-sufficiency; Consumption arbitrage	no	no
(Kloess and Zach, 2014)	Pumped Hydro; CAES; Hydrogen; Methane	Sell at high prices (trading); Buy at low prices (trading)	Trading arbitrage	no	no
(Lai and McCulloch, 2017)	Batteries	Buy at low prices (consumption)	Self-sufficiency	no	no
(Lin and Wu, 2017)	Batteries	Sell at high prices (trading); Buy at low prices (trading)	Trading arbitrage	yes	no
(Linssen, Stenzel and Fleer, 2017)	Batteries	Buy at low prices (consumption)	Self-sufficiency	yes	no
(Locatelli, Palerma and Mancini, 2015)	Pumped Hydro; CAES	Provide short-term frequency restoration (T&D); Provide long-term frequency restoration (T&D); Sell at high prices (trading); Buy at low prices (trading)	Short-term frequency restoration; Long-term frequency restoration; Trading arbitrage	no	yes
(Loisel, 2012)	CAES	Provide short-term frequency restoration (trading); Provide long-term frequency restoration (trading); Sell at high prices (trading); Buy at low prices (trading)	Frequency Control; Trading arbitrage	no	yes
(Lombardi and Schwabe, 2017)	Batteries	Meet selling forecast (production); Shave demand peaks (consumption); Buy at low prices (consumption)	Production forecast; Peak shaving; Self-sufficiency	yes	yes
(Madlener and Latz, 2013)	CAES	Provide long-term frequency restoration (production); Sell at high prices (trading); Buy at low prices (trading)	Frequency control; Trading arbitrage	yes	yes
(McHenry, 2012)	Batteries	Buy at low prices (consumption)	Self-sufficiency	no	no
(Merei *et al.*, 2016)	Batteries	Buy at low prices (consumption)	Self-sufficiency	no	no
(Mulder *et al.*, 2013)	Batteries	Buy at low prices (consumption)	Self-sufficiency	yes	no
(Papaefthymiou and Papathanassiou, 2014)	Pumped Hydro	Sell at high prices (trading); Buy at low prices (trading); Shave demand peaks (production)	Trading arbitrage; Peak shaving	yes	yes
(Parra and Patel, 2016)	Batteries	Buy at low prices (consumption); Shave demand peaks (consumption)	Self-sufficiency; Peak shaving	no	yes
(Parra *et al.*, 2017)	Batteries	Shave demand peaks (consumption); Buy at low prices (consumption)	Self-sufficiency; Peak shaving; Consumption arbitrage	no	yes
(Reuter *et al.*, 2012)	Pumped Hydro	Sell at high prices (trading); Buy at low prices (trading)	Trading arbitrage	no	no
(Rudolf and Papastergiou, 2013)	Batteries	Sell at high prices (trading); Buy at low prices (trading)	Trading arbitrage	no	no
(Staffell and Rustomji, 2016)	Batteries	Sell at high prices (trading); Buy at low prices (trading); Provide long-term frequency restoration (T&D)	Trading arbitrage; Long-term frequency restoration	no	yes

(Stephan *et al.*, 2016)	Batteries	Provide frequency containment (T&D); Provide short-term frequency restoration (T&D); Provide long-term frequency restoration (T&D); Shave supply / demand peaks (T&D); Shave demand peaks (consumption); Buy at low prices (consumption)	Frequency containment; Short-term frequency restoration; Long-term frequency restoration; Peak shaving; Peak shaving; Self-sufficiency	no	yes
(van der Stelt, AlSkaif and van Sark, 2018)	Batteries	Buy at low prices (consumption)	Self-sufficiency; Consumption arbitrage	no	no
(Yu and Foggo, 2017)	Batteries	Sell at high prices (trading); Buy at low prices (trading); Provide frequency containment (T&D); Provide short-term frequency restoration (T&D); Provide long-term frequency restoration (T&D)	Trading arbitrage; Frequency containment; Short-term frequency restoration; Long-term frequency restoration	yes	yes
(Zafirakis *et al.*, 2013)	Pumped Hydro; CAES	Meet selling forecast (production); Save generation capacity reserve (production)	Production forecast; Peak shaving	no	yes
(Zheng, Meinrenken and Lackner, 2015)	Batteries; Flywheels; Magnetic Storage; Pumped Hydro; CAES; Capacitors	Shave demand peaks (consumption); Buy at low prices (consumption)	Peak shaving; Consumption arbitrage	yes	yes
(Zucker and Hinchliffe, 2014)	Batteries	Sell at high prices (trading); Buy at low prices (trading); Buy at low prices (consumption)	Trading arbitrage; Self-sufficiency	no	yes

Supplemental Figures

	Business Model	Mechanical									Thermal			Chemical								
		Flywheel			Pumped Hydro			CAES			Thermal			Supercapacit.			Batteries			Hydrogen		
	**Trading**	∞	$	#	∞	$	#	∞	$	#	∞	$	#	∞	$	#	∞	$	#	∞	$	#
CA	Frequency containment	3	-	-	2	1.0	1	2	1.0	1	2	-	-	2	-	-	3	0.0	1	2	-	-
CA	Short-term frequency restoration	3	-	-	2	1.0	1	2	0.5	2	2	-	-	2	-	-	3	-	-	3	-	-
CA	Long-term frequency restoration	3	-	-	3	1.0	1	2	0.5	2	3	-	-	2	-	-	3	0.0	1	3	-	-
CA	Trading forecast	2	-	-	2	-	-	3	-	-	3	-	-	1	-	-	3	-	-	3	-	-
PA	Trading arbitrage	2	0.0	2	3	0.7	9	3	0.6	12	3	-	-	2	0.0	2	3	0.3	10	3	0.0	1
	**Production**	∞	$	#	∞	$	#	∞	$	#	∞	$	#	∞	$	#	∞	$	#	∞	$	#
CA	Frequency containment	3	-	-	2	-	-	2	-	-	2	-	-	2	-	-	3	1.0	1	2	-	-
CA	Short-term frequency restoration	3	0.0	1	2	0.5	2	2	0.0	1	2	-	-	2	0.0	1	3	0.0	1	3	-	-
CA	Long-term frequency restoration	3	0.0	1	3	0.0	1	2	0.5	2	3	-	-	2	0.0	1	3	0.0	1	3	-	-
CA	Production forecast	2	-	-	3	0.0	1	3	0.3	3	3	-	-	1	-	-	3	1.0	1	3	-	-
CA	Schedule flexibility	2	-	-	3	-	-	3	-	-	3	-	-	1	-	-	3	-	-	3	-	-
ID	Voltage control	3	-	-	1	-	-	2	-	-	2	-	-	3	-	-	3	-	-	2	-	-
ID	Backup energy	2	-	-	2	-	-	2	-	-	3	-	-	1	-	-	3	-	-	3	-	-
ID	Black start energy	3	-	-	2	-	-	2	-	-	3	-	-	2	-	-	3	-	-	3	-	-
ID	Peak Shaving	2	-	-	3	0.8	4	3	0.0	1	3	-	-	1	-	-	3	1.0	1	3	-	-
	**T&D**	∞	$	#	∞	$	#	∞	$	#	∞	$	#	∞	$	#	∞	$	#	∞	$	#
CA	Frequency containment	3	-	-	2	-	-	2	0.5	2	2	-	-	2	-	-	3	0.3	3	2	-	-
CA	Short-term frequency restoration	3	-	-	2	0.0	1	2	0.3	3	2	-	-	2	-	-	3	0.3	3	3	-	-
CA	Long-term frequency restoration	3	-	-	3	0.0	1	2	0.3	3	3	-	-	2	-	-	3	0.3	4	3	-	-
CA	Black start energy	3	-	-	2	-	-	2	0.0	1	3	-	-	2	-	-	3	-	-	3	-	-
ID	Voltage control	3	-	-	1	1.0	1	2	1.0	1	2	-	-	3	-	-	3	-	-	2	-	-
ID	Peak shaving	2	-	-	3	-	-	3	-	-	3	-	-	1	-	-	3	0.0	2	3	0.0	1
	**Consumption**	∞	$	#	∞	$	#	∞	$	#	∞	$	#	∞	$	#	∞	$	#	∞	$	#
CA	Frequency containment	3	-	-	2	-	-	2	-	-	2	-	-	2	-	-	3	-	-	2	-	-
CA	Short-term frequency restoration	3	-	-	2	-	-	2	-	-	2	-	-	2	-	-	3	0.0	1	3	-	-
CA	Long-term frequency restoration	3	-	-	3	-	-	2	-	-	3	-	-	2	-	-	3	-	-	3	-	-
CA	Peak shaving	2	1.0	1	2	1.0	1	2	1.0	1	3	-	-	2	1.0	1	3	0.5	8	3	-	-
ID	Voltage control	3	-	-	1	-	-	2	-	-	2	-	-	3	-	-	3	-	-	2	-	-
ID	Backup energy	2	-	-	2	-	-	2	-	-	3	-	-	1	-	-	3	0.0	1	3	-	-
PA	Self-sufficiency	2	-	-	2	1.0	1	3	1.0	1	3	-	-	2	-	-	3	0.4	20	3	-	-
PA	Consumption arbitrage	2	-	-	2	1.0	1	3	1.0	1	3	-	-	2	-	-	3	0.2	9	3	-	-

CA Cost avoidance   ID Investment deferral   PA Price arbitrage

Supplemental Figure S1, Related to Figure 2. Technology match and profitability of business models for energy storage. The first column (∞) indicates the matching of business models with storage technologies, the second column ($) the profitability and the third column (#) the number of studies that examine the profitability of a match.

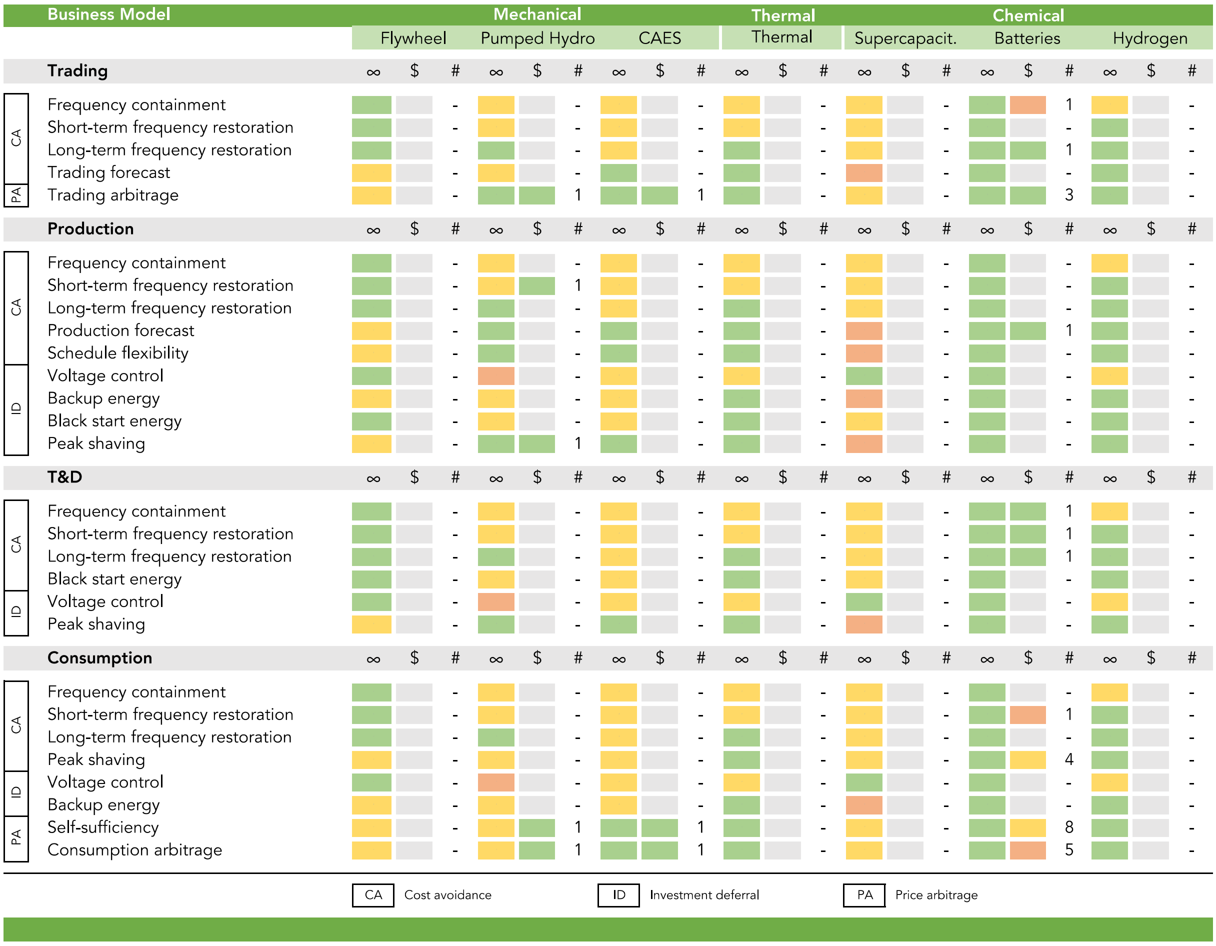

Supplemental Figure S2, Related to Figure 2. Profitability of business models for energy storage (2017-2019). The first column (∞) indicates the matching of business models with storage technologies, the second column ($) the profitability and the third column (#) the number of studies that examine the profitability of a match.

References

Akhil, A. A. *et al.* (2013) *DOE/EPRI 2013 Electricity Storage Handbook in Collaboration with NRECA*. doi: SAND2013-5131.

Aneke, M. and Wang, M. (2016) 'Energy storage technologies and real life applications – A state of the art review', *Applied Energy*. Elsevier Ltd, 179, pp. 350–377. doi: 10.1016/j.apenergy.2016.06.097.

Arabkoohsar, A. *et al.* (2015) 'Thermo-economic analysis and sizing of a PV plant equipped with a compressed air energy storage system', *Renewable Energy*. Elsevier Ltd, 83, pp. 491–509. doi: 10.1016/j.renene.2015.05.005.

Barton, J. P. and Infield, D. G. (2004) 'Energy Storage and Its Use With Intermittent Renewable Energy', *IEEE Transactions on Energy Conversion*, 19(2), pp. 441–448. doi: 10.1109/TEC.2003.822305.

Battke, B. and Schmidt, T. S. (2015) 'Cost-efficient demand-pull policies for multi-purpose technologies - The case of stationary electricity storage', *Applied Energy*, 155, pp. 334–348. doi: 10.1016/j.apenergy.2015.06.010.

Beaudin, M. *et al.* (2010) 'Energy storage for mitigating the variability of renewable electricity sources: An updated review', *Energy for Sustainable Development*, 14(4), pp. 302–314. doi: http://dx.doi.org/10.1016/j.esd.2010.09.007.

Berrada, A. and Loudiyi, K. (2016) 'Operation, sizing, and economic evaluation of storage for solar and wind power plants', *Renewable and Sustainable Energy Reviews*. Elsevier, 59, pp. 1117–1129. doi: 10.1016/j.rser.2016.01.048.

Berrada, A., Loudiyi, K. and Zorkani, I. (2016) 'Valuation of energy storage in energy and regulation markets', *Energy*. Elsevier Ltd, 115, pp. 1109–1118. doi: 10.1016/j.energy.2016.09.093.

Berrada, A., Loudiyi, K. and Zorkani, I. (2017) 'Profitability, risk, and financial modeling of energy storage in residential and large scale applications', *Energy*, 119, pp. 94–109. doi: 10.1016/j.energy.2016.12.066.

Bertuccioli, L. *et al.* (2014) *Study on development of water electrolysis in the EU*, *Fuel Cells and hydrogen Joint Undertaking*.

Bortolini, M., Gamberi, M. and Graziani, A. (2014) 'Technical and economic design of photovoltaic and battery energy storage system', *Energy Conversion and Management*. Elsevier Ltd, 86, pp. 81–92. doi: 10.1016/j.enconman.2014.04.089.

de Bosio, F. and Verda, V. (2015) 'Thermoeconomic analysis of a Compressed Air Energy Storage (CAES) system integrated with a wind power plant in the framework of the IPEX Market', *Applied Energy*. Elsevier Ltd, 152, pp. 173–182. doi: 10.1016/j.apenergy.2015.01.052.

Bradbury, K., Pratson, L. and Patiño-Echeverri, D. (2014) 'Economic viability of energy storage systems based on price arbitrage potential in real-time U.S. electricity markets', *Applied Energy*. Elsevier Ltd, 114, pp. 512–519. doi: 10.1016/j.apenergy.2013.10.010.

Braff, W. A., Mueller, J. M. and Trancik, J. E. (2016) 'Value of storage technologies for wind and solar energy', *Nature Climate Change*, 6(10), pp. 964–969. doi: 10.1038/NCLIMATE3045.

Broneske, G. and Wozabal, D. (2016) 'How Do Contract Parameters Influence the Economics of Vehicle-to-Grid?', *Manufacturing & Service Operations Management*. INFORMS, 19(1), pp. 1–34. doi: 10.1287/msom.2016.0601.

Chazarra, M. *et al.* (2018) 'Economic viability of pumped-storage power plants participating in the secondary regulation service', *Applied Energy*. Elsevier, 216(October 2017), pp. 224–233. doi: 10.1016/j.apenergy.2018.02.025.

Chen, H. *et al.* (2009) 'Progress in electrical energy storage system: A critical review', *Progress in Natural Science*. National Natural Science Foundation of China and Chinese Academy of Sciences, 19(3), pp. 291–312. doi: 10.1016/j.pnsc.2008.07.014.

Cho, J., Jeong, S. and Kim, Y. (2015) 'Commercial and research battery technologies for electrical energy storage applications', *Progress in Energy and Combustion Science*. Elsevier Ltd, 48, pp. 84–101. doi:

10.1016/j.pecs.2015.01.002.

Comello, S. and Reichelstein, S. (2019) 'The emergence of cost effective battery storage', *Nature Communications*. Springer US, 10(1), p. 2038. doi: 10.1038/s41467-019-09988-z.

Das, T., Krishnan, V. and McCalley, J. D. (2015) 'Assessing the benefits and economics of bulk energy storage technologies in the power grid', *Applied Energy*. Elsevier Ltd, 139, pp. 104–118. doi: 10.1016/j.apenergy.2014.11.017.

Denholm, P. *et al.* (2010) *The role of energy storage with renewable electricity generation*.

Dufo-López, R. and Bernal-Agustín, J. L. (2015) 'Techno-economic analysis of grid-connected battery storage', *Energy Conversion and Management*, 91, pp. 394–404. doi: 10.1016/j.enconman.2014.12.038.

EPRI (2010) *Electric Energy Storage Technology Options: A White Paper Primer on Applications, Costs and Benefits*.

Eyer, J. and Corey, G. (2010) *Energy Storage for the Electricity Grid : Benefits and Market Potential Assessment Guide*. doi: SAND2010-0815.

Eyer, J. M., Iannucci, J. J. and Corey, G. P. (2004) *Energy Storage Benefits and Market Analysis Handbook - A Study for the DOE Energy Storage Systems Program*.

Fares, R. L. and Webber, M. E. (2017) 'The impacts of storing solar energy in the home to reduce reliance on the utility', *Nature Energy*, 2(2), p. 17001. doi: 10.1038/nenergy.2017.1.

Ferreira, H. L. *et al.* (2013) 'Characterisation of electrical energy storage technologies', *Energy*. Elsevier Ltd, 53, pp. 288–298. doi: 10.1016/j.energy.2013.02.037.

Fleer, J. *et al.* (2018) 'Techno-economic evaluation of battery energy storage systems on the primary control reserve market under consideration of price trends and bidding strategies', *Journal of Energy Storage*. Elsevier Ltd, 17, pp. 345–356. doi: 10.1016/j.est.2018.03.008.

Fuchs, G. *et al.* (2012) *Technology Overview on Electricity Storage - Overview on the potential and on the deployment perspectives of energy storage technologies*.

Gallo, A. B. *et al.* (2016) 'Energy storage in the energy transition context: A technology review', *Renewable and Sustainable Energy Reviews*, 65, pp. 800–822. doi: 10.1016/j.rser.2016.07.028.

Gough, R. *et al.* (2017) 'Vehicle-to-grid feasibility: A techno-economic analysis of EV-based energy storage', *Applied Energy*. Elsevier Ltd, 192, pp. 12–23. doi: 10.1016/j.apenergy.2017.01.102.

Härtel, P. *et al.* (2016) 'Cost assessment of storage options in a region with a high share of network congestions', *Journal of Energy Storage*, 8, pp. 358–367. doi: 10.1016/j.est.2016.05.010.

Hartmann, B., Divényi, D. and Vokony, I. (2018) 'Evaluation of business possibilities of energy storage at commercial and industrial consumers – A case study', *Applied Energy*. Elsevier, 222(March), pp. 59–66. doi: 10.1016/j.apenergy.2018.04.005.

Hoppmann, J. *et al.* (2014) 'The economic viability of battery storage for residential solar photovoltaic systems -- A review and a simulation model', *Renewable and Sustainable Energy Reviews*, 39, pp. 1101–1118. doi: 10.1016/j.rser.2014.07.068.

Johnston, L. *et al.* (2015) 'Methodology for the economic optimisation of energy storage systems for frequency support in wind power plants', *Applied Energy*. Elsevier Ltd, 137, pp. 660–669. doi: 10.1016/j.apenergy.2014.09.031.

Kaschub, T., Jochem, P. and Fichtner, W. (2016) 'Solar energy storage in German households: profitability, load changes and flexibility', *Energy Policy*, 98, pp. 520–532. doi: 10.1016/j.enpol.2016.09.017.

Khalilpour, K. R. and Vassallo, A. (2016) 'Technoeconomic parametric analysis of PV-battery systems', *Renewable Energy*. Elsevier Ltd, 97, pp. 757–768. doi: 10.1016/j.renene.2016.06.010.

Kloess, M. and Zach, K. (2014) 'Bulk electricity storage technologies for load-leveling operation – An economic assessment for the Austrian and German power market', *International Journal of Electrical Power & Energy Systems*, 59, pp. 111–122. doi: http://dx.doi.org/10.1016/j.ijepes.2014.02.002.

Koohi-Kamali, S. *et al.* (2013) 'Emergence of energy storage technologies as the solution for reliable

operation of smart power systems: A review', *Renewable and Sustainable Energy Reviews*, 25, pp. 135–165. doi: 10.1016/j.rser.2013.03.056.

Lai, C. S. and McCulloch, M. D. (2017) 'Levelized cost of electricity for solar photovoltaic and electrical energy storage', *Applied Energy*. Elsevier Ltd, 190, pp. 191–203. doi: 10.1016/j.apenergy.2016.12.153.

Lin, B. and Wu, W. (2017) 'Economic viability of battery energy storage and grid strategy: A special case of China electricity market', *Energy*. Elsevier Ltd, 124, pp. 423–434. doi: 10.1016/j.energy.2017.02.086.

Linssen, J., Stenzel, P. and Fleer, J. (2017) 'Techno-economic analysis of photovoltaic battery systems and the influence of different consumer load profiles', *Applied Energy*. Elsevier Ltd, 185(2017), pp. 2019–2025. doi: 10.1016/j.apenergy.2015.11.088.

Locatelli, G., Palerma, E. and Mancini, M. (2015) 'Assessing the economics of large Energy Storage Plants with an optimisation methodology', *Energy*. Elsevier Ltd, 83, pp. 15–28. doi: 10.1016/j.energy.2015.01.050.

Loisel, R. (2012) 'Power system flexibility with electricity storage technologies: A technical-economic assessment of a large-scale storage facility', *International Journal of Electrical Power and Energy Systems*. Elsevier Ltd, 42(1), pp. 542–552. doi: 10.1016/j.ijepes.2012.04.058.

Lombardi, P. and Schwabe, F. (2017) 'Sharing economy as a new business model for energy storage systems', *Applied Energy*. Elsevier Ltd, 188, pp. 485–496. doi: 10.1016/j.apenergy.2016.12.016.

Madlener, R. and Latz, J. (2013) 'Economics of centralized and decentralized compressed air energy storage for enhanced grid integration of wind power', *Applied Energy*, 101, pp. 299–309. doi: http://dx.doi.org/10.1016/j.apenergy.2011.09.033.

McHenry, M. P. (2012) 'Are small-scale grid-connected photovoltaic systems a cost-effective policy for lowering electricity bills and reducing carbon emissions? A technical, economic, and carbon emission analysis', *Energy Policy*. Elsevier, 45, pp. 64–72. doi: 10.1016/j.enpol.2012.01.036.

Merei, G. *et al.* (2016) 'Optimization of self-consumption and techno-economic analysis of PV-battery systems in commercial applications', *Applied Energy*. Elsevier Ltd, 168, pp. 171–178. doi: 10.1016/j.apenergy.2016.01.083.

Mulder, G. *et al.* (2013) 'The dimensioning of PV-battery systems depending on the incentive and selling price conditions', *Applied Energy*. Elsevier Ltd, 111, pp. 1126–1135. doi: 10.1016/j.apenergy.2013.03.059.

Palizban, O. and Kauhaniemi, K. (2016) 'Energy storage systems in modern grids - Matrix of technologies and applications', *Journal of Energy Storage*. Elsevier Ltd, 6, pp. 248–259.

Papaefthymiou, S. V. and Papathanassiou, S. A. (2014) 'Optimum sizing of wind-pumped-storage hybrid power stations inisland systems', *Renewable Energy*. Elsevier Ltd, 64, pp. 187–196. doi: 10.1016/j.renene.2013.10.047.

Parra, D. *et al.* (2017) 'Optimum community energy storage for renewable energy and demand load management', *Applied Energy*. Elsevier Ltd, 200, pp. 358–369. doi: 10.1016/j.apenergy.2017.05.048.

Parra, D. and Patel, M. K. (2016) 'Effect of tariffs on the performance and economic benefits of PV-coupled battery systems', *Applied Energy*. Elsevier Ltd, 164(2016), pp. 175–187. doi: 10.1016/j.apenergy.2015.11.037.

Reuter, W. H. *et al.* (2012) 'Investment in wind power and pumped storage in a real options model', *Renewable and Sustainable Energy Reviews*. Elsevier Ltd, 16(4), pp. 2242–2248. doi: 10.1016/j.rser.2012.01.025.

Rudolf, V. and Papastergiou, K. D. (2013) 'Financial analysis of utility scale photovoltaic plants with battery energy storage', *Energy Policy*, 63, pp. 139–146. doi: http://dx.doi.org/10.1016/j.enpol.2013.08.025.

Sayer, J. H., Eyer, J. and Brown, R. S. (2007) *Guide to Estimating Benefits and Market Potential for Electricity Storage in New York*.

Schoenung, S. (2001) *Characteristics and Technologies for Long- vs. Short-Term Energy Storage*. doi: 10.2172/780306.

de Sisternes, F. J., Jenkins, J. D. and Botterud, A. (2016) 'The value of energy storage in decarbonizing the electricity sector', *Applied Energy*. Elsevier Ltd, 175, pp. 368–379. doi: 10.1016/j.apenergy.2016.05.014.

Staffell, I. and Rustomji, M. (2016) 'Maximising the value of electricity storage', *Journal of Energy Storage*.

Elsevier Ltd, 8, pp. 212–225. doi: 10.1016/j.est.2016.08.010.

van der Stelt, S., AlSkaif, T. and van Sark, W. (2018) 'Techno-economic analysis of household and community energy storage for residential prosumers with smart appliances', *Applied Energy*. Elsevier, 209(October 2017), pp. 266–276. doi: 10.1016/j.apenergy.2017.10.096.

Stephan, A. *et al.* (2016) 'Limiting the public cost of stationary battery deployment by combining applications', *Nature Energy*, 1(7), p. 16079. doi: 10.1038/nenergy.2016.79.

Yu, N. and Foggo, B. (2017) 'Stochastic valuation of energy storage in wholesale power markets', *Energy Economics*. Elsevier B.V., 64, pp. 177–185. doi: 10.1016/j.eneco.2017.03.010.

Zafirakis, D. *et al.* (2013) 'Modeling of financial incentives for investments in energy storage systems that promote the large-scale integration of wind energy', *Applied Energy*, 105, pp. 138–154. doi: 10.1016/j.apenergy.2012.11.073.

Zheng, M., Meinrenken, C. J. and Lackner, K. S. (2015) 'Smart households: Dispatch strategies and economic analysis of distributed energy storage for residential peak shaving', *Applied Energy*. Elsevier Ltd, 147, pp. 246–257. doi: 10.1016/j.apenergy.2015.02.039.

Zucker, A. and Hinchliffe, T. (2014) 'Optimum sizing of PV-attached electricity storage according to power market signals – A case study for Germany and Italy', *Applied Energy*, 127, pp. 141–155. doi: http://dx.doi.org/10.1016/j.apenergy.2014.04.038.
